# Development and Relevance of Hypercapnia in COPD

**DOI:** 10.1155/2021/6623093

**Published:** 2021-02-22

**Authors:** Chirag Dave, Simon Wharton, Rahul Mukherjee, Bandar M. Faqihi, Robert A. Stockley, Alice M. Turner

**Affiliations:** ^1^University Hospitals Birmingham NHS Foundation Trust, Birmingham, UK; ^2^Institute of Applied Health Research, University of Birmingham, Birmingham, UK

## Abstract

**Background:**

Identification of patients who may become hypercapnic, or develop acidotic hypercapnic respiratory failure (AHRF), is important in chronic obstructive pulmonary disease (COPD) to avoid hospital admission and select patients for use of home NIV. This study aimed to identify factors associated with presence and development of hypercapnia.

**Methods:**

1224 patients, 637 with COPD and 587 with alpha 1 antitrypsin deficiency (AATD), from 4 previously established patient cohorts, were included in cross-sectional analyses of hypercapnia (PaCO_2_ ≥ 6.5 kPa or 48.8 mmHg), focusing on phenotypic features of COPD and mortality. Longitudinal associations of rising PaCO_2_ were also assessed. A second cohort of 160 COPD patients underwent sleep studies and 1-year follow-up, analysing in a similar way, incorporating additional information from their sleep studies if appropriate.

**Results:**

Hypercapnia was 15 times more common in usual COPD than AATD (*p* < 0.01) after adjustment for baseline differences by regression. Independent predictors of hypercapnia in COPD included FEV_1_ and current use of oxygen; these variables, together with lack of emphysema, explained 11% of variance in CO_2_. Increasing PaCO_2_ also associated with higher risk of death (*p*=0.03). 44/160 patients exhibited sleep disordered breathing. The sleep study cohort also showed an association of low FEV_1_ with hypercapnia. Prior hospital admission for AHRF was also clinically significant, being a feature of almost double the number of hypercapnic patients in both test and sleep study COPD cohorts.

**Conclusion:**

Lower FEV_1_ and prior AHRF are the main associations of hypercapnia in COPD, which carries a poor prognosis, particularly worsening over time.

## 1. Introduction

The most important function of the respiratory system is to maintain blood oxygenation and exhale carbon dioxide, when impaired respiratory failure defined as significant hypoxia with or without hypercapnia can develop. In chronic obstructive pulmonary disease (COPD), both hypoxia and hypercapnia may occur; in over 12 000 patients reviewed as part of the European COPD audit, the median PaCO_2_ was 5.8 kPa (43.5 mmHg) and PaO_2_ was 7 kPa (52.5 mmHg) on hospital admission [1]. In the UK, 35% of all COPD admissions have respiratory failure, and 44% have an elevated PaCO_2_, suggesting that acidotic hypercapnic respiratory failure (AHRF) is relatively frequent [2].

AHRF is a well-defined complication of COPD, and noninvasive ventilation (NIV) is an effective treatment in such patients [3]. NIV has also been used nightly at home to manage chronic respiratory failure in COPD [4] and is now a commonly used treatment in severe disease. An episode of AHRF in the context of COPD is a poor prognostic marker, with only 50% of patients alive one year later [5]. Early research suggested that stable-state hypercapnia was also a key prognostic factor in patients with COPD [6] with a hazard ratio of 2.90 for death at 1 year when PaCO_2_ ≥ 6 kPa (45 mmHg) measured on admission for an acute exacerbation of COPD (AECOPD) [7]. Furthermore, in 5-year follow-up data comparing those with type 1 respiratory failure, those who had acute type 2 respiratory failure that resolved (type 2.1) had a better long-term survival compared to those where it persisted (type 2.2) [8]. Consistent with this epidemiological evidence, the largest treatment benefit of home NIV is seen in patients with persistent hypercapnia [9, 10].

A number of factors in COPD have been reported to contribute to hypercapnia, including breathing pattern [11], inspiratory muscle weakness [11], hyperinflation [12], continued smoking [12], and FEV_1_ below 36% [13]. However, in each of these studies, the overall proportion of variance in carbon dioxide level explicable by the reported variables was low. Identification of patients who are likely to become hypercapnic, or to develop AHRF, is important in COPD if we are to avoid the deleterious consequences of hospital admission and select patients for use of home NIV in a timely manner. We hypothesized that inadequate characterisation might be the reason that previous studies had been unable to construct an adequate predictive model for hypercapnia. In particular, we felt that more data on emphysema, comorbidity, and sleep parameters would be important missing variables. The current study aimed to identify COPD patients predisposed to hypercapnia through a combined approach within COPD cohorts, including a large group with detailed COPD phenotyping, enriched for those at risk of hypercapnia by including those with prior AHRF or severe disease (defined by current LTOT use), and a newly recruited group who also underwent detailed sleep studies.

## 2. Methods

### 2.1. COPD Phenotype Cohort

There were four sources of patients, three with usual COPD and one with COPD related to AATD. The first with usual COPD was the Birmingham Heartlands Hospital NIV cohort, a population that had required NIV for AHRF described elsewhere [14]; patients with alternative pathologies (e.g., obesity-related hyperventilation) were excluded. Patients were recruited at admission and had blood gas analyses approximately 3 months after discharge. The second usual COPD cohort included all patients from the West Midlands COPD cohort, described previously [15]. The third usual COPD cohort was those in a long-term oxygen (LTOT) clinic [16]. In these cohorts, the presence of emphysema was determined by the appearance on computed tomography (CT) scan. If present, its location was defined as upper and lower zone, or both; quantitative analysis was not possible due to differences in scanners and scan protocol.

The fourth cohort was patients with AATD known to the Birmingham AATD registry who had at least one stable-state blood gas; procedures for assessment are described elsewhere [17]. Rate of decline per year in blood gas parameters was calculated in those that had at least 4 values over 3 years, using methods described in our previous work pertaining to FEV_1_ decline [18]. The presence and zone of emphysema was determined by appearance and densitometry (where possible), as described previously [19]. In the combined cohort, survival was determined at the end of 2014, using medical/GP records. Local ethical approval was given for all research cohorts, and patients gave informed consent.

### 2.2. Sleep Study Cohort

Patients with clinically diagnosed COPD, confirmed by postbronchodilator spirometry, and with at least 1 hospitalisation for COPD in the last 12 months were recruited prospectively from University Hospitals Birmingham NHS Foundation Trust between August 2014 and 2015; the study was ethically approved, and all patients gave informed consent. We enriched the cohort for those at risk of hypercapnia by approaching all patients with a previous admission for AHRF. Patients on home NIV or CPAP were excluded. All patients were then assessed in the stable state with a full medical history, Epworth sleepiness score (ESS) and CAT questionnaires, spirometry, blood gases, and limited channel polysomnography (ResMed Embletta Gold) at baseline, and this process is repeated, without the sleep study, at follow-up 12 months later. Where chest CT scans were available, these were reviewed in the same manner as the previous cohorts. All sleep studies were analysed only if there was >240 minutes of sleep recorded and were of good quality; those that did not meet these criteria were repeated. Studies were reported according to AASM scoring manual 2.2 by a single researcher (CD) and an independent sleep technician.

### 2.3. Statistical Analysis

Data were analysed in SPSS version 24.0, and results were considered statistically significant when *p* < 0.05. Data normality was determined visually and by the Kolmogorov–Smirnov test; normally and nonnormally distributed variables are expressed as mean (standard error) or median (interquartile range), respectively. Of 21 studies of home NIV vs usual care in our systematic review, 12 studies defined hypercapnia as PCO_2_ >6.5 kPa (48.8 mmHg) [4]; hence, this was taken to define hypercapnia in our study. In the COPD phenotype cohort, univariate analyses were conducted and variables were then taken forward, in a stepwise manner if *p* < 0.05 to multiple logistic regression models assessing CO_2_, presence of hypercapnia, or rising CO_2_ during follow-up. Cox-regression analysis was used to assess the impact of hypercapnia and rising CO_2_ on mortality, using a stepwise approach to determine covariates. Those variables that appeared to relate to outcome were then tested in the sleep study cohort, in whom independent multivariable analyses of hypercapnia, rising CO_2_, and mortality were also conducted, which incorporated additional information from their sleep studies if appropriate. Linear regression was used to assess CO_2_ as a quantitative outcome, both to align to prior research assessing it in this way and give an indication of what proportion of variance was explicable by included variables. Independent associations of obstructive sleep apnoea (OSA) were also sought using similar techniques.

## 3. Results

### 3.1. COPD Phenotype Cohort

1224 patients, 637 with COPD and 587 with AATD, were included. Characteristics of the COPD patients are shown in Table 1, stratified by presence of AHRF in COPD. This was not relevant in AATD since no patients had reported this event previously. Characteristics of the AATD group are shown in Supplementary Table 1. The prevalence of hypercapnia was much higher in usual COPD than AATD (*p* < 0.01), with usual COPD patients 14.82 (95% CI: 6.67–32.92, *p* < 0.01) times more likely to be hypercapnic after adjustment for baseline differences by regression. Consequently, we decided to analyse the AATD and COPD groups separately and present the AATD results only in the supplement allowing to focus on usual COPD in the main manuscript. In usual COPD, there were significant associations between hypercapnia and reduced FEV_1_ (but not with DLco or volumatic measures of gas trapping), use of LTOT, lower PO_2_, higher HCO_3_, and base excess (Table 1). Independent predictors of hypercapnia in COPD in the multivariate model included FEV_1_ and current use of LTOT (Table 2); these variables explained 11% of variance in CO_2_. In multivariate analyses, the risk of hypercapnia was doubled per 1 kPa (7.5 mmHg) lower PaO_2_ (odds ratio (OR): 2.05, 95% confidence interval: 1.23–3.33, *p* < 0.01) and current smoking status conferred an OR for hypercapnia of 9.69 (95% CI: 2.85–33.08, *p* < 0.01); together these accounted for 28% of variance in CO_2_. Emphysema zone did not relate to hypercapnia in either group, whether examined visually or (in AATD) by densitometry (458 scans; *p* > 0.1).

Follow-up blood gases were not carried out often in the COPD groups; thus, the number of patients in whom deteriorating CO_2_ could be assessed was relatively small, and no clinically significant associations were seen in univariate models. Although serial blood gases were available in all AATD patients, few developed hypercapnia and no meaningful regression analysis were possible due to small numbers (Supplementary Table 2).

**Figure 1:**
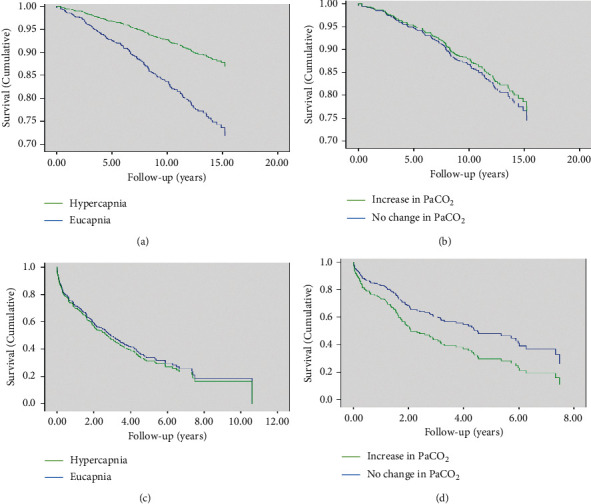
Cox regression for mortality in AATD and COPD. (a) AATD, hypercapnia and survival, (b) AATD, increasing CO_2_ and survival, (c) COPD, hypercapnia and survival, (d) COPD, increasing CO_2_ and survival.

Survival curves for COPD and AATD populations, stratified by the presence of hypercapnia or rising PaCO_2_, are shown in Figure 1. Hypercapnia did not influence mortality within the usual COPD group (OR: 0.93 (95% CI: 0.70–1.25)). However, the presence of an increasing stable-state PaCO_2_ was a predictor of mortality; patients were 1.65 (95% CI: 1.06–2.56, *p*=0.03) times more likely to die if they had an increasing PaCO_2_.

### 3.2. Sleep Study Cohort

176 patients were recruited, of whom 16 were excluded, 8 due to spirometry not being diagnostic of COPD, 3 due to current CPAP use, 2 due to home NIV use, and 3 due to inability to obtain an adequate sleep study after 2 attempts. Of the 160 remaining, 94 had never had AHRF and 66 had required NIV for AHRF on a previous hospital admission; their clinical characteristics are shown in Table 3 stratified by presence of prior AHRF. Thirty-four (21%) were current smokers, and the remainder all were ex-smokers. Comorbidities were common; 66 (41%) had hypertension, 33 (20%) had documented vascular disease (ischaemic heart disease, atrial fibrillation, and cerebral or peripheral vascular disease), and 16 (10%) had osteoporosis. 108/160 patients (68%) had a CT scan available for analysis; no patient had lower zone dominant disease. Of the variables independently associated with hypercapnia in the phenotype cohort, this was replicated only for FEV_1_ (*p* < 0.05). Notably, hypercapnia was generally less common in this cohort and LTOT use less frequently (expected due to nature of recruitment of test cohort).

Forty-four patients exhibited sleep disordered breathing, 43 having OSA and 1 having central apnoeic events; amongst these, 17 had ESS>10 (i.e., obstructive sleep apnoea hypopnoea syndrome, OSAHS), and all of whom were then offered CPAP. The only independent association of OSA in this group was baseline CO_2_ (Supplementary Tables 3 and 4). Few differences in the most recent NIV admission were seen; only a small increase was seen in time on NIV and length of hospital stay, although neither was significant in multivariate analysis (Supplementary Tables 5 and 6). Nocturnal hypoventilation also appeared to differ between those who had or had not had AHRF, as shown by time during which O_2_ saturations were<90%. Addition of OSA to the multivariate model for hypercapnia did not improve its positive predictive value which may reflect few OSA cases in hypercapnic patients (*n* = 4, Supplementary Table 2).

No patient was lost to follow-up, although 15 deaths occurred, 5 in those who had never had AHRF, and 10 in those with prior AHRF. Although the average CO_2_ level did not appear markedly different at follow-up, there were a higher number of patients with hypercapnia in both those who had AHRF and those who had not. Baseline hypercapnia also significantly associated with death at 1 year (11/15 deaths if hypercapnic, 10/145 who were not; *p* < 0.01, OR: 1.67 (1.45–2.89), Supplementary Figure 1).

## 4. Discussion

Our study has shown that patients with AATD are at low risk of hypercapnia compared to usual COPD patients. The main novel findings of our work are that longitudinal change in CO_2_ may be more important than hypercapnia itself when considering risk of death and that hyperinflation (irrespective of its cause) could be more relevant to development of hypercapnia than emphysema itself.

### 4.1. Which COPD Patients Become Hypercapnic?

We chose to study defined hypercapnia, rather than just PaCO_2_ variance, as we felt this would be more applicable to clinical decision-making. The results confirmed previous work assessing PaCO_2_ [12], concurring with previous evidence that poor lung function is associated with hypercapnia. In the usual COPD cohort, a number of markers of severe disease (lower FEV_1_ or DLCOpp, higher FRCpp, and current LTOT prescription) were associated with hypercapnia. Historically, spirometry has been poorly associated with blood gas parameters, but PaCO_2_ does rise more sharply after FEV_1_ falls below 40% predicted [13]. Almost half our hypercapnic patients were under this threshold compared to only 33% of eucapnic patients. This suggests that overall alveolar ventilation is adequate up to a threshold, beyond which it becomes difficult to maintain.

Hypercapnia tended to be less likely if there was emphysema on CT. This was to some extent unexpected as previous studies linked hyperinflation, a measure of air trapping, and potentially therefore an indirect measure of emphysema, to higher PaCO_2_ [6, 20]. Conversely, the classical cachectic “pink puffer” with emphysema has long been thought more likely to have type 1 not type 2 respiratory failure [21]. Studies have suggested that the location of emphysema could have a direct physiological effect because of differences in the mechanical forces present, as lower zone emphysema directly impairs diaphragmatic motion [22]; however, in our data, emphysema location had no impact on hypercapnia, and moreover, in AATD (where most patients have lower zone dominant disease), hypercapnia was markedly less common. Although visual CT scan inspection might be considered a crude method to quantify the extent of the emphysema, even densitometry, which was assessed for many of the AATD patients, did not reveal an association of emphysema zone or severity with hypercapnia, suggesting that any impact on the diaphragm alone is insufficient to cause alveolar hypoventilation. Direct measurement of diaphragmatic function might have strengthened the evidence supporting this hypothesis, but this was not possible in our study.

Whilst emphysema did not associate positively with the presence of hypercapnia, we acknowledge that a quantitative CT analysis could not be done in the COPD cohort; thus, it may be relevant that other surrogate markers of air trapping (lower DLCOpp, increasing hyperinflation as measured by FRCpp) were associated with hypercapnia. Such air trapping could occur with airways disease or emphysema. It has been well documented that NIV in stable hypercapnic COPD patients reduces hyperinflation amongst other parameters [23], which in turn increases daytime PaO_2_ and reduces daytime PaCO_2_. This suggests that amount of hyperinflation independent of presence or amount of emphysema may be more of an influence on PaCO_2_. Other potential unmeasured influences relevant to the relationship between emphysema and hypercapnia include the degree of VQ matching, which could differ by affected zone, and contributions from dynamic rather than static hyperinflation.

### 4.2. What Is the Prognosis with Hypercapnia?

Although hypercapnic patients have a short-term mortality risk reported by others [8] and confirmed in our data (albeit not as high a risk as earlier studies have found) if survival is present beyond 2 years and certainly over 10 years, there does not seem to be any obvious difference compared to nonhypercapnic COPD patients [24]. A more recent study showed that 5.12% of a eucapnic COPD cohort progressed to develop hypercapnia (PaCO_2_>6 kPa or 45 mmHg) during long-term follow-up [25] and 13.3% of these had reversible hypercapnia following discharge from hospital. Thus, eucapnic COPD may actually represent a subphenotype and not just simply an “early stage” of COPD that will eventually develop hypercapnia. In the same cohort of Chinese patients, median survival was longer in the eucapnic vs hypercapnic groups (6.5 vs 5 years, *p*=0.02) [25]. With the advent of home NIV as a treatment proven to reduce mortality [9] and hospital admissions [10] in COPD, it is possible that prognosis will continue to improve, but only if these high-risk patients are identified early for treatment.

### 4.3. Which COPD Patients Should Have a Sleep Study?

OSA was more likely in patients with a history of AHRF, but this did not increase the likelihood of OSAHS, and thus such patients would not necessarily present to medical services with symptoms of sleepiness. Furthermore, even though mean PaCO_2_ was slightly higher in the OSAHS group (5.68 vs 5.4), it did not reach statistical significance; this implies that identifying OSAHS in COPD patients would require widespread screening, since the CO_2_ would be an insufficiently sensitive distinguishing feature to adopt as a case finding approach. Such patients also appeared to have delayed weaning from acute NIV, although this was also not significant in multivariate analysis due to the small differences in ventilation duration driven by strict protocols for weaning in our unit [26] which may have limited our ability to detect any relationship. The value of routine sleep studies in the non-AHRF population was questionable given that significant problems occurred in only 15% of patients.

The PaCO_2_ is a well-established physiological difference in patients with COPD-OSA overlap and COPD alone. Two hundred and thirteen consecutive patients compared in one study had a mean PaCO_2_ difference of 5.94 kPa (44.5 mmHg) vs 5.28 kPa (39.6 mmHg) [27] although why this occurred remains unclear. Regression analysis within the same cohort could only partially explain the occurrence of hypercapnia by the combination of reduced respiratory function and being overweight, which suggests that there is clearly a multifactorial element to this phenomenon. In our cohort, no associations with anthropometric features were seen, but it is notable that few of our patients exhibited very high BMI.

Overnight hypoxia in overlap syndrome has been shown to be more severe compared to COPD alone as both COPD and OSA cause desaturations [28]. However, in a large cohort of nonobese, severe COPD patients, the number of patients needing supplemental oxygen did not differ between OSA and overlap syndrome patients [29]. Other studies have found mean oxygen saturations at similar levels between COPD and overlap groups [30]. Our data showed that time spent under 90% saturations during the sleep study was significantly more in the OSA patients. However, there was no difference in mean saturations, which suggests that there are more extreme hypoxia events when the COPD patients seem to be more at risk of having an apnoeic or hypopnoeic episode. Whilst simple overnight oximetry to screen for OSA within COPD appeared attractive based on this feature, it was not statistically significant in the regression model seeking independent predictors of OSA, so time spent under 90% saturations is unlikely to be a useful screening tool. We, therefore, propose an algorithm to detect COPD-OSA overlap whereby any patient who has AHRF and remains hypercapnic at discharge should have repeat blood gases at 4 weeks, and if they remain hypercapnic, a limited channel sleep study should be done in order to formally calculate AHI.

### 4.4. What Do Our Data Tell Us about Natural History of COPD?

The two cohorts studied comprised one with a very broad range of disease severity (the AATD group) and some that were more homogeneous (COPD groups); in part, this is related to the way that cohorts were recruited, in which all COPD patients presented routinely to respiratory services with symptoms whereas some AATD patients could have been diagnosed whilst asymptomatic as a result of family screening. In this broader cohort, we observed less hypercapnia and even over long periods of follow-up and little progression in terms of blood CO_2_ level. However, in the more homogeneous, generally more severe COPD cohort deterioration in this parameter was notable and related to survival. Taken together with our previous results, we suggest that hyperinflation develops in severe disease and contributes to development of hypercapnia only in advanced disease. Once it has developed, further deterioration is indicative of end-stage disease with a high risk of death, which is why rising CO_2_ in this group was associated with death. These people might be the group most relevant for home NIV and active palliative care.

### 4.5. Strengths and Limitations

The large cohorts studied, assessing a wider range of phenotypic features and outcomes, and robustly assessing COPD-OSA overlap in the replication cohort are strengths. Although we focussed on patients at high risk of hypercapnia (LTOT use and previous episodes of AHRF), there was good representation of disease severities; a fifth of hypercapnic patients had an FEV_1_ >50% predicted. Furthermore, the inclusion of the AATD population allowed inclusion of those with mild disease, yet at risk of rapid progression. However, there were clear limitations; the measure of “increasing PaCO_2_” is not validated, and we did not use a comorbidity score (e.g., Charlson) to adjust for this in our multivariable analyses. Finally, the AATD cohort may be prone to acquisition bias, since those with hypercapnia may have poorer survival, or less ability to travel for assessment; nevertheless, the large number of very severe COPD patients seen suggests that this is not a major factor.

## 5. Conclusions

Lower FEV_1_ and prior AHRF are the main associations of stable-state hypercapnia in COPD, which carries a poor prognosis, particularly if it worsens over time in the stable state. Whilst sleep disordered breathing does occur and is clinically important, conducting a sleep study is likely only to be valuable in patients who are persistently hypercapnic in the stable state.

## Figures and Tables

**Table 1:** Characteristics of the COPD cohort.

	All COPD *N* = 637	Comparison based on prior AHRF in COPD		
		No AHRF *N* = 323	AHRF *N* = 314	*p*
Age (years)	74 (66–80)	75 (68–82)	65 (73–78)	**<0.01**
Sex (male)	278 (43.6)	148 (45.8)	140 (44.59)	0.75
Pack years	40 (33–56)	40 (32–55)	40 (33–56)	0.59
Current smokers	126 (19.8)	49 (15.2)	77 (24.5)	**0.02**
Hypercapnia	233 (36.6)	73 (22.6)	145 (46.18)	**<0.01**
BMI	25.5 (213–32.5)	24.6 (21.3–32.0)	26·7 (21.4–33.6)	0.28
FEV_1_	0.9 (0.65–1.26)	0.98 (0.72–1.43)	0·82 (0.59–1.12)	**<0.01**
FEV_1_pp	41 (41–55)	47 (34–60)	36 (28–49)	**<0.01**
FVCpp	73 (57–90)	78 (63–94)	69 (51–85)	**<0.01**
DLCOpp	48.9 (38.9–73.3)	60.9 (55.1–66.7)	43.6 (36.2–64.9)	**<0.01**
RVpp	104.35 (89.5–123.4)	100 (88.03–108.93)	112 (93.4–134.25)	0.09
PaO_2_	7.9 (7.1–8.7)	7.9 (7.2–8.7)	7.8 (7.0–8.6)	0.08
PaCO_2_	5.9 (4.9–6.9)	5.5 (4.7–6.4)	6.4 (5.4–7.5)	**<0.01**
HCO_3_	28.4 (26.2–31.5)	27.5 (25.6–30.1)	29 (26.7–32.8)	**<0.01**
BE	3.7 (1.5–6.5)	3.4 (1.2–5.7)	4.3 (2.3–7.2)	**<0.01**
On LTOT	304 (47.7)	146 (45.2)	158 (50.3)	0.2
Emphysema	284 (44.6)	177 (54.8)	142 (45.2)	**0.04**
Upper lobe emphysema	113 (39.8)	58 (51.3)	55 (48.7)	0.21
Lower lobe emphysema	14 (4.9)	12 (85.7)	2 (14.3)	0.12
No zonal predominance	157 (55.3)	89 (56.69)	68 (43.3)	0.79
LZVI-UZVI	—	—	—	—
Bronchiectasis	101 (15.9)	50 (15.5)	51 (16.2)	0·79
Change in FEV_1_/ml/year	−54 (−160-23.5)	−65.5 (−172.8-8)	−49 (−143.5−32.5)	0·33
Follow-up (years)	1.33 (0.31–2.65)	1.30 (0.31–2.56)	0.31 (1.34–2.87)	0·62
Death	415 (65.1)	192 (59.4)	223 (71.02)	**<0.01**

Data are shown as mean (SE) or median (IQR) for quantitative measures, and *n* (%) for binary outcomes, which are further denoted by italic text.

**Table 2:** Multiple regression analysis assessing hypercapnia.

	OR	*p* value
FEV_1_ pp	0.98 (0.97–0.99)	**<0·01**
Presence of emphysema	0.72 (0.50–1.05)	**0·09**
Use of LTOT	1.74 (1.19–2.53)	**<0·01**
DLCOpp	0.92 (0.89–0.94)	**<0.01**
FRCpp	1.17 (1.09–1.25)	**<0.01**

The ORs shown represent the effect of increasing FEV_1_ by 1% of predicted, and DLCO, by 1% predicted, having emphysema *v* not, using LTOT *v* not, increasing age by 1 year, and current smoking *v* not. *p* = percent predicted.

**Table 3:** Characteristics of the sleep study cohort at baseline and 1 year.

	No AHRF (*n* = 94)	AHRF (*n* = 66)	*p* value
Age (years)	65.4 (63.4–67.5)	67.6 (65.4–69.8)	0.16
Sex (male)	50 (53.2)	34 (51.5)	0.83
Weight (kg)	71.0 (60.1–84.4)	76.8 (71.2–82.3)	0.47
BMI	26.6 (23.2–31.7)	29.1 (27.1–31.1)	0.31
pH	7.4 (7.4–7.4)	7.4 (7.4–7.4)	**0.04**
PaO_2_	9.2 (8.9–9.7)	8.5 (8.1–8.8)	**<0.01**
PaCO_2_	5.0 (4.5–5.6)	5.7 (5.5–5.9)	**<0.01**
HCO_3_^−^	25.2 (24.0–26.5)	27.4 (26.2–28.7)	**<0.01**
BE	0.6 (−1.1-2.5)	2.35 (1.2–4.9)	**<0.01**
Hypercapnia	7 (7.4)	13 (19.7)	**0.02**
CAT	23.5 (21.8–25.2)	22.7 (20.5–25.0)	0.5
Epworth sleep score	9.0 (5.0–14.0)	8.00 (4.0–12.0)	0.2
Bronchiectasis (% of those with CT)	13/68 (19.1)	5/40 (12.5)	0.37
Emphysema	56/68 (82.35)	30/40 (75)	0.59
LTOT	10/94 (10.6)	10/66 (15.2)	0.40
Pack years	45.0 (30.0–60.0)	48.5 (38.0–64.3)	0.12
FEV_1_	1.4 (0.9–1.8)	0.9 (0.7–1.5)	**<0.01**
FEV_1_ % predicted	56.5 (52.2–60.9)	43.77 (39.55–47.99)	**<0.01**
FVC	2.7 (2.6–2.9)	2.3 (2.1–2.5)	**<0.01**
FVC % predicted	86.5 (72.0–100.0)	76.1 (71.2–81.0)	**<0.01**
FEV_1_/FVC	0.5 (0.5–0.6)	0.5 (0.4–0.5)	**0.02**
OSA on sleep study	14 (14.9)	29 (43.9)	**<0.01**
AHI	2.4 (0.6–4.6)	3.7 (0.5–8.8)	0.09
ODI	5.6 (3.3–10.1)	7.5 (4.5–14.1)	0.08
Mean oxygen sats during sleep study	90.4 (87.7–92.5)	89.6 (85.6–92.6)	0.26
Time under 90%	12.0 (1.6–52.3)	23.1 (3.5–96.1)	**0.04**
Death at 12 months	5/94 (5.3)	10/66 (15.2)	0.35
Follow-up PaCO_2_	4.9 (4.5–5.6)	4.9 (4.5–5.4)	0.66

Data are shown as mean (SE) or median (IQR) for quantitative measures, and *n* (%) for binary outcomes, which are further denoted by italic text. CAT = COPD assessment test, LTOT = long-term oxygen therapy, AHI = apnoea hyponoea index, and ODI = oxygen desaturation index.

## Data Availability

Data are available upon reasonable request.

## Supplementary Materials

Supplementary Materials Supplementary Table 1: characteristics of AATD cohort. Supplementary Table 2: characteristics of patients whose arterial CO_2_ worsened over time. Supplementary Table 3: characteristics of COPD patients with or without OSA. Supplementary Table 4: independent associations of COPD-OSA overlap. Supplementary Table 5: impact of undiagnosed OSA on prior hospital admission with AHRF. Supplementary Table 6: independent associations of undiagnosed OSA. Supplementary Figure 1: hypercapnia and survival in the sleep cohort.
