# Supplementary material for: Development and Relevance of Hypercapnia in COPD
Source: Can Respir J. 2021 Feb 22;2021:6623093. doi: 10.1155/2021/6623093 (PMC7920710; doi:10.1155/2021/6623093)
Supplement: Supplementary Materials — Supplementary Table 1: characteristics of AATD cohort. Supplementary Table 2: characteristics of patients whose arterial CO2 worsened over time. Supplementary Table 3: characteristics of COPD patients with or without OSA. Supplementary Table 4: independent associations of COPD-OSA overlap. Supplementary Table 5: impact of undiagnosed OSA on prior hospital admission with AHRF. Supplementary Table 6: independent associations of undiagnosed OSA. Supplementary Figure 1: hypercapnia and survival in the sleep cohort. [file 6623093.f1.docx]

# Supplementary material

| **n(%)** | **All AATD**  **N=587** |  |
| --- | --- | --- |
|  |  |  |
| **Age** | 50.8 (43.5-57.6) |  |
| ***Sex (male)*** | *332 (56.6)* |  |
| **Pack years** | 15 (2.5-25.5) |  |
| ***Current smokers*** | 51 (8.7) |  |
| ***Hypercapnia*** | *13 (2.2)* |  |
| **BMI** | 25 (22.4-27.9) |  |
| **FEV_1_** | 1.23 (0.8-2.0) |  |
| **FEV_1_pp** | 44.3 (28.7-70.1) |  |
| **FVCpp** | 99.23 (75.4-115.7) |  |
| **DLCOpp** | 71.1 (54.2-92.07) |  |
| **RVpp** | 122.64 (98.77-154.98) |  |
| **PaO_2_** | 9.10 (8.40-10.10) |  |
| **PaCO_2_** | 4.80 (4.40-5.10) |  |
| **HCO_3_** | 24 (22-25) |  |
| **BE** | 0.55 (-1-2) |  |
| ***On LTOT*** | *55 (9.4)* |  |
| ***Emphysema*** | 375 (63.88) |  |
| ***Upper lobe emphysema*** | *18 (3.0)* |  |
| ***Lower lobe emphysema*** | *390 (66.5)* |  |
| ***No zonal predominance*** | *179 (30.5)* |  |
| **LZVI-UZVI** | 13.45 (4-22.71) |  |
| ***Bronchiectasis*** | *116 (26.6)* |  |
| **Change in FEV_1_/ml/year** | -42.4 (-74.0-15.0) |  |
| **Follow-up (years)** | 11.22 (7.50-14.11) |  |
| ***Death*** | *159 (27.10)* |  |
| Supplementary table 1: Characteristics of AATD cohort | | |

|  | **COPD** | | | **AATD** | | |
| --- | --- | --- | --- | --- | --- | --- |
|  | **Stable PaCO_2_**  **n=61** | **Increasing PaCO_2_**  **n=68** | **p** | **Stable PaCO_2_**  **n=338** | **Increasing PaCO_2_**  **n=149** | **p** |
| **Age** | 75 (67-81) | 70 (64-77) | **0·02** | 51·3 (44·1-58·7) | 50·79 (42·3-57·1) | **0·04** |
| ***Sex (male)*** | *34 (55·7)* | *30 (44·1)* | *0·19* | *201 (56·2)* | *79 (53·0)* | *0·19* |
| **Pack years** | 38 (30-52·5) | 40 (34·25-50) | 0·41 | 15 (1·6-15) | 15 (3·8-25·8) | 0·63 |
| ***Current smokers*** | *10 (16·7)* | *10 (14·71)* | *0·88* | *33 (9·8)* | *14 (9·4)* | *0·9* |
| ***Hypercapnia*** | *35 (57·4)* | *21 (31·9)* | ***<0·01*** | *8 (2.37)* | *0 (0)* | *0·06* |
| **BMI** | 27·8 (22·7-34·1) | 25·4 (21·1-37·4) | 0·7 | 22·9 (22·6-27·8) | 25·3 (22·3-28·1) | 0·94 |
| **FEV_1_** | 0·97 (0·67-1·36) | 0·94 (0·7-1·32) | 0·84 | 1·3 (0·8-1·9) | 1·2 (0·8-2·1) | 0·76 |
| **FEV_1_pp** | 41 (31-54) | 43 (30-54) | 0·88 | 45·9 (28·9-72·7) | 44·1 (33·2-69·9) | 0·90 |
| **FVCpp** | 73 (60-93) | 73 (56-98) | 0·88 | 101·3 (76.6-116.0) | 100·5 (82·8-115·7) | 0·94 |
| **DLCOpp** | 46.7 (38.9-67.8) | 74.4 (59.0-89.8) | **<0.01** | 72·2 (55·4-91·8) | 70·4 (52.0-93·7) | 0·72 |
| **RVpp** | 104.5 (89.56-123.4) | 88.8 (70.1-107.4) | 0.09 | 120·7(96·4-150·8) | 123·2 (99·3-150·1) | 0·44 |
| **RV/TLC(%)** | 22.1 (18.2-27.1) | 18.9 (13.2-24.6) | 0.08 | 72·2 (55·4-91·8) | 70·4 (52.0-93·7) | 0·72 |
| **PaO_2_** | 7.3 (6.7-8.0) | 7.4 (6.9-8.3) | 0·51 | 8·4 (9·2-10·1) | 9·1 (8·4-9·9) | 0·60 |
| **PaCO_2_** | 6·7 (5·7-7·4) | 5·9 (4·7-6·7) | **<0·01** | 4·8 (4·4-5·1) | 4·8 (4·4-5·1) | 0·98 |
| **HCO_3_** | 29·1 (26·5-32·5) | 28 (25·6-30·9) | **0·03** | 24 (22-25) | 24 (22-25) | 0·85 |
| **BE** | 4·2 (2·4-7·5) | 3·4 (2·1-5) | **0·03** | 0·4 (-0.7-2) | 0·8 (-1·0-1·8) | 0·59 |
| ***Emphysema*** | *30 (49·2)* | *43 (63·2)* | *0·11* | *219 (64·8)* | *98 (65·8)* | *0·27* |
| **Change in FEV_1_/ml/year** | -72·5(-217·5--7·2) | -59·5 (-160--2) | 0·44 | -46·39 (-80·01--16·69) | -36·00 (-55·18--11·50) | 0·17 |
| ***≥1 episode NIV*** | *33 (54·10)* | *35 (51·50)* | *0·77* | *0* | *0* | *-* |
| **Follow-up (years)** | 2·04 (1·43-3·24) | 2·64 (1·73-4·37) | 0·07 | 11·4 (7·8-14·2) | 11·4 (7·6-14·1) | 0·90 |
| ***Death*** | *29 (47·5)* | *34 (50)* | *0·78* | *85 (25·15)* | *32 (21·48)* | *0·38* |

Supplementary table 2: Characteristics of patients whose arterial CO2 worsened over time

**Results**

**AATD phenotype cohort**

In AATD, hypercapnia was uncommon, thus reducing power to detect associations, nevertheless a significant relationship was seen with lower FEV_1_ and current smoking. Neither hypercapnia (OR 2·36 (95% CI 0·57-9·83) p=0·24), nor increasing PaCO_2_ (OR 1·10 (95% CI 0·72-1·68), p=0·65) were statistically associated with increased risk of death in AATD, even though the curves separated markedly for hypercapnia, likely reflecting small numbers.

**Analyses of longitudinal changes in CO2**

#### Usual COPD

Univariate analyses of the 129 (20·25%) usual COPD patients with serial blood gases available are shown in supplementary table 1. These patients were younger (69.5 vs 75, p<0.01), had a lower PaCO_2_ to begin with (5.9kPA vs 6.69kPa, p<0.01) and had a greater change in PaO_2_/year (-0.17kPa vs 0.08kPa/year, p<0.02). COPD patients were more likely to be hypercapnic to begin with, in the group with no increasing PaCO_2_ 57.4% vs 31.90 (p<0.01). BMI and smoking status had no discernible difference noted with p values of 0.88 and 0.7 respectively. There was no difference in FEV_1_pp (41 vs 43, p=0.88), FVCpp (both 73%, p=0.88). The difference in the FEV_1_ decline per year was not too different either (-72.5 vs -59.5, p=0.44). There was no difference noted in the presence of emphysema or its location. The only more detailed lung function parameter that had any significant difference was DLCOpp which was significantly lower in the stable CO_2_ group at 46.7% vs 74.43% in the increasing group (p<0.01).

#### AATD

In total, 487 (83%) AATD patients had serial PaCO_2_ values available. In total 30.60% of patients had increasing PaCO_2_ over time. No patients with an increasing PaCO_2_ were hypercapnic to begin with (p=0.06). Patients tended to be younger (50.79 vs 51.32 year, p=0.04), but otherwise had no discernible difference in basic spirometry (FEV_1 pp_ 44.10 vs 45.95, p=0.90) or more detailed testing: KCOpp 63.21 vs 65.55, p=0.31. BMI, smoking status and presence of emphysema did not differ at all, with p-values of 0.94, 0.9 and 0.27 respectively.

#### Multiple regression analysis

In the usual COPD population the independent predictors in multivariate modelling were age with an OR of 0.94 (0.88-1.0, p=0.07), DLCOpp OR of 1.03 (1.00-1.07, p=0.08) and PaCO_2_ with an OR of 0.78 (0.45-1.34, p=0.37). Only 1.65% variance could be explained with R^2^ value of 0.16. No meaningful regression model could be generated using the statistically significant variables in univariate analysis for the AATD patients with increasing PaCO_2_.

|  | **No OSA (n=117)** | **Yes OSA (n=43)** | **p value** |
| --- | --- | --- | --- |
| Age (years) | 67.03 (65.15-68.70) | 64.71 (61.96-67.45) | 0.62 |
| Sex n male(%) | 61/117 | 23/43 | 0.88 |
| Weight (kg) | 74.00 (62.00-85.60) | 73.00 (67.18-78.82) | 0.39 |
| BMI | 26.95 (23.01-32.50) | 27.82 (25.75—29.91) | 0.69 |
| pH | 7.42 (7.40-7.45) | 7.41 (7.40-7.42) | 0.07 |
| PaO_2_ | 8.93 (8.65-9.21) | 8.69 (8.28-9.09) | 0.36 |
| PaCO_2_ | 5.35 (4.65-5.70) | 5.63 (5.34-5.92) | **0.02** |
| HCO_3_^-^ | 26.20 (24.50-28.2) | 28.24 (25.59-30.91) | **0.04** |
| BE | 1.30 (-0.02-3.40) | 1.90 (1.17-2.63) | 0.53 |
| CAT | 25.00(16.00-29.00) | 23.98 (21.29-26.66) | 0.55 |
| ESS | 4.00 (4.00-12.00) | 10 (5.00-14.50) | 0.14 |
| LTOT (%) | 11/117 (9.40) | 9/43 (20.93) | **0.04** |
| Pack years | 45.00 (35.00-60.00) | 46.00 (36.00-68.00) | 0.62 |
| FEV_1_ | 1.18 (80.79-1.66) | 1.08 (0.74-1.68) | 0.47 |
| FEV_1_% predicted | 49.76 (35.00-64.00) | 50.60 (44.15-57.05) | 0.89 |
| FVC | 2.53 (2.38-2.68) | 2.57 (2.30-2.83) | 0.63 |
| FVC % predicted | 82.00 (65.00-96.00) | 85.64 (78.50-90.78) | 0.84 |
| Mean sats | 94.10 (86.50-96.60) | 89.10 (87.80-92.60) | 0.08 |
| Central events | 0.5 (0.5-10..4) | 0.4 (0.3-1.1) | 0.89 |
| Time under 90% (%) | 8.80 (1.00-65.20) | 23.10 (3.30-99.45) | **0.04** |
| Hypercapnia (%) | 17/117 (14.53) | 4/43 (9.30) | 0.39 |
| Death at 12 months (%) | 8/117 (6.83) | 3/43 (6.98) | 0.98 |

Supplementary table 3: Characteristics of COPD patients with or without OSA

| **Variable** | **OR** | **P value** |
| --- | --- | --- |
| OPD PaCO_2_ | 1.65 (1.06-2.57) | **0.03** |
| LTOT | 2.56 (1.15-1.89) | 0.06 |
| % time spent under 90% | 1 (0.99-1.01) | 0.5 |

Supplementary table 4: Independent associations of COPD-OSA overlap

Logistic regression analysis for new OSA cases, bought forward from the univariate analysis shown in supplementary table 1 if p<0.05. The OR value shows the effect of increasing PaCO2 by 1, of having LTOT vs not, and effect of increasing by 1% the time spent under 90% saturations.

|  | **No OSA (n=37)** | **New OSA (n=29)** | **P value** |
| --- | --- | --- | --- |
| Initiation pH | 7.27 (7.19-7.33) | 7.24 (7.17-7.32) | 0.4 |
| Initiation PaCO_2_ | 8.42 (6.90-13.02) | 8.98 (6.86-14.01) | 0.5 |
| Initiation PaO_2_ | 10.30 (7.78-12.44) | 10.98 (6.99-11.58) | 0.3 |
| Initiation HCO_3_^-^ | 27.38 (25.67-29.40) | 26.87 (24.65-27.04) | 0.6 |
| Days on NIV | 5.2 | 6.3 | **0.02** |
| LOS | 9.4 (3-16) | 11.5 (3-23.5) | **0.02** |
| IPAP | 14.55 (12.5-22.1) | 16.78 (12.5-23.05) | 0.21 |
| EPAP | 5.71 (4-12) | 5.45 (4-14) | 0.89 |
| Discharge PaO_2_ | 7.28 (6.6-9.45) | 7.45 (7.1-10.01) | 0.45 |
| Hypercapnia on discharge | 8 | 10 | 0.38 |
| Discharge PaCO_2_ | 6.6 (4.9-7.0) | 6.9 (5.5-7.4) | 0.12 |
| Discharge HCO_3_^-^ | 30.10 (28.70-33.45) | 31.30 (26.74-34.67) | 0.04 |
| Discharge BE | 3.10 (1.90-4.54) | 3.99 (-0.09-5.19) | 0.31 |
| Time under 90% (%) | 15.42 (3.23-44.45) | 34.45 (28.56-99.45) | **0.04** |
| OPD pH | 7.43 (7.43-7.45 | 7.42 (7.42-7.43) | 0.76 |
| OPD PaO_2_ | 8.82 (6.64-10.56) | 8.62 (6.4-11.01) | 0.7 |
| OPD PaCO_2_ | 6.3 (4.9-6.6) | 6.7 (5.5-7.4) | 0.67 |
| OPD HCO_3_ | 26.87 (24.7-32.03) | 30.15 (28.04-33.45) | 0.05 |
| Hypercapnia (%) | 6 | 4 | 0.45 |

Supplementary table 5: Impact of undiagnosed OSA on prior hospital admission with AHRF

| **Variable** | **OR** | **P value** |
| --- | --- | --- |
| Days on NIV | 0.82 (0.60-1.11) | 0.21 |
| % time spent under 90% | 1.00 (0.98-1.02) | 0.66 |

Supplementary table 6: Independent associations of undiagnosed OSA

Logistic regression analysis for new OSA cases, bought forward from the univariate analysis shown in supplematary table 3. The OR value shows the effect of increasing days on NIV by 1 and effect of increasing by 1% the time spent under 90% saturations.


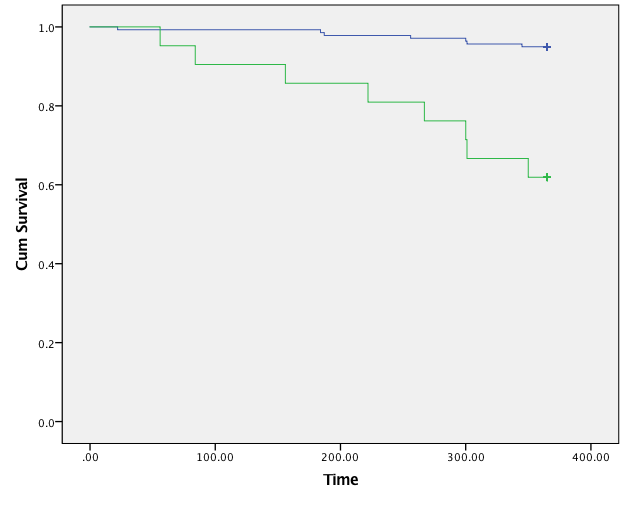


PCO_2_ < 6.5kPa

PCO_2_ > 6.5kPa

Supplementary figure 1: Hypercapnia and survival in the sleep cohort
